# The blowfly *Chrysomya latifrons* inhabits fragmented rainforests, but shows no population structure

**DOI:** 10.1007/s00442-023-05333-w

**Published:** 2023-02-11

**Authors:** Nathan J. Butterworth, James F. Wallman, Nikolas P. Johnston, Blake M. Dawson, Joshua Sharp-Heward, Angela McGaughran

**Affiliations:** 1 School of Biological Sciences, Monash University, Clayton, VIC 3800 Australia; 2 Faculty of Science, University of Technology Sydney, Ultimo, NSW 2007 Australia; 3 Department of Ecology and Biogeography, Faculty of Biological and Veterinary Sciences, Nicolaus Copernicus University in Toruń, 87-100 Toruń, Poland; 4 Centre for Sustainable Ecosystem Solutions, School of Earth, Atmospheric and Life Sciences, University of Wollongong, Wollongong, NSW 2522 Australia; 5 Te Aka Mātuatua - School of Science, University of Waikato, Private Bag 3105, Hamilton, 3240 New Zealand

**Keywords:** Genetic structure, Ecology, Diptera, Rainforest, Habitat fragmentation

## Abstract

Climate change and deforestation are causing rainforests to become increasingly fragmented, placing them at heightened risk of biodiversity loss. Invertebrates constitute the greatest proportion of this biodiversity, yet we lack basic knowledge of their population structure and ecology. There is a compelling need to develop our understanding of the population dynamics of a wide range of rainforest invertebrates so that we can begin to understand how rainforest fragments are connected, and how they will cope with future habitat fragmentation and climate change. Blowflies are an ideal candidate for such research because they are widespread, abundant, and can be easily collected within rainforests. We genotyped 188 blowflies (*Chrysomya latifrons*) from 15 isolated rainforests and found high levels of gene flow, a lack of genetic structure between rainforests, and low genetic diversity – suggesting the presence of a single large genetically depauperate population. This highlights that: (1) the blowfly *Ch. latifrons* inhabits a ~ 1000 km stretch of Australian rainforests, where it plays an important role as a nutrient recycler; (2) strongly dispersing flies can migrate between and connect isolated rainforests, likely carrying pollen, parasites, phoronts, and pathogens along with them; and (3) widely dispersing and abundant insects can nevertheless be genetically depauperate. There is an urgent need to better understand the relationships between habitat fragmentation, genetic diversity, and adaptive potential–especially for poorly dispersing rainforest-restricted insects, as many of these may be particularly fragmented and at highest risk of local extinction.

**Supplementary Information:**

The online version contains supplementary material available at 10.1007/s00442-023-05333-w.

## Introduction

Due to climate change and deforestation, rainforests are becoming increasingly fragmented islands of endemic biodiversity (Bowman 2000; Lens et al. 2002 Laurance et al. 2017) that are highly susceptible to biodiversity loss (Williams et al. 2003; Mariani et al. 2019; Nolan et al. 2020). To make informed conservation decisions for rainforests and their inhabitants, we must understand how these habitats are fragmented, and how this affects the genetic patterns and ecological processes of their flora and fauna.

Species that inhabit fragmented rainforests can vary significantly in their extent of population genetic structure and diversity – spanning a continuum from well-connected to fragmented populations and low to high genetic diversity (Leung et al. 1994; Brown et al. 2004; Milá et al. 2009; Brito and Arias 2010; Woltmann et al. 2012; Sadanandan and Rheindt 2015). These unique genetic responses to fragmentation result from species-specific physiologies, dispersal capacities, resource requirements, and habitat continua, as well as the landscape structure of the fragments they inhabit (i.e., the presence and number of corridors between adjacent fragments) (Leung et al. 1994; Callens et al. 2011; Woltmann et al. 2012). The consequences of these differences are twofold: (1) certain species will suffer greater genetic and ecological consequences from habitat fragmentation; while (2) other species will play greater roles in maintaining connectivity between fragments. Understanding these species-specific responses is central to quantifying and mitigating further habitat fragmentation under a changing climate.

While attention has focused on many vertebrate animals experiencing anthropogenic threats (Shapcott 2000; Shoo et al. 2005; Mac Nally et al. 2009; Moritz et al. 2005; Martínez-Ramos et al. 2016), invertebrates have been largely neglected (Ellwood and Foster 2004; Snaddon 2008; Wardhaugh et al. 2012; Taylor et al. 2018; New 2018). Of all animals, invertebrates are the largest contributors to the function and biodiversity of rainforest ecosystems (Ewers et al. 2015; Griffiths et al. 2017) and perform crucial roles in maintaining habitat connectivity by promoting gene flow in their associated plants and parasites (Jabis et al. 2011; DiBlasi et al. 2018). Despite this, we have a very limited understanding of the population structure of most rainforest invertebrates (Radespiel and Bruford 2014), including whether there is connectivity/gene flow among geographically disconnected fragments, and which species and populations require conservation priority. There is an urgent need to remedy this, as studies around the world are beginning to identify significant changes in invertebrate populations, with habitat fragmentation and climate change as key drivers (Cardoso et al. 2020; Samways et al. 2020; Legge et al. 2021; Marsh et al. 2021).

Australian rainforests are naturally fragmented due to the unique geological and climatic history of the continent (Bowman 2000). However, many rainforests have also been further fragmented by human processes since European colonisation (Bowman 2000) as well as due to increasing rates of wildfires (Legge et al. 2021; Trouvé et al. 2021). Importantly, these highly fragmented rainforests hold a substantial proportion of the continent’s total invertebrate biodiversity (Kitching et al. 1993; Yeates et al. 2003; Stork and Grimbacher 2006). As such, there is a need to understand how invertebrates are distributed throughout these rainforests, determine whether there is gene flow between populations, and identify which species and populations are less likely to cope with sudden changes to climate or habitat structure (Kelly 2019; Razgour et al. 2019) and which contribute most to maintaining connectivity between habitat fragments. However, given the overwhelming diversity of rainforest invertebrates, it is not currently feasible to comprehensively assess the genetic responses of every species to habitat fragmentation. The simplest solution is to begin studying species that inhabit fragmented rainforests but are broadly distributed throughout them, can easily disperse between them, and can be reliably collected. Such species should show generally high levels of genetic connectivity between habitat fragments, providing a baseline understanding of how rainforest fragments are connected, and which are most isolated, to inform how they might cope with further fragmentation.

Blowflies (Diptera: Calliphoridae) are well-suited to this approach because they are widespread throughout rainforests, extremely abundant, and easy to capture in the wild (Norris 1959; Badenhorst and Villet 2018; Butterworth et al. 2020). Blowflies are also highly vagile (Norris 1959; Tsuda et al. 2009) and many species exhibit wide dietary breadths – opportunistically feeding on a range of resources, including vertebrate and invertebrate carrion, dung, decaying plant matter, and pollen (Dear 1985; Brodie et al. 2015). As such, blowflies are likely some of the most capable invertebrate dispersers and can be expected to play important roles in maintaining connectivity between even the most highly isolated habitat fragments.

**Fig. 1:**
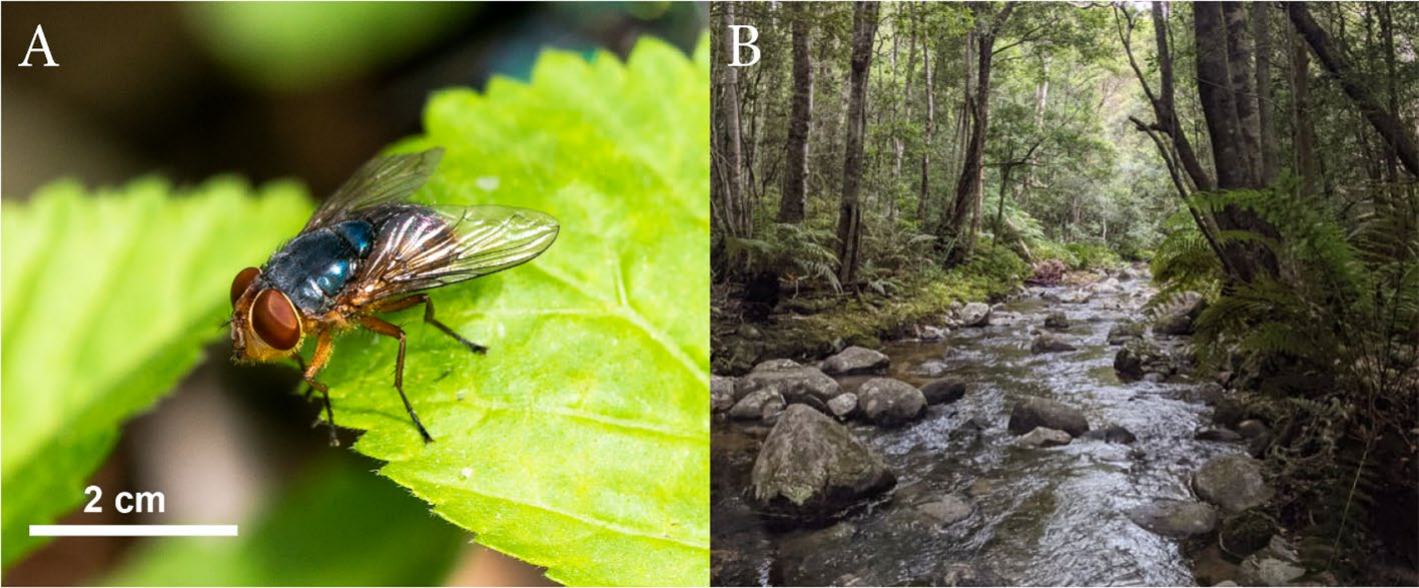
**A** The blowfly *Chrysomya latifrons* which are endemic to southeast Australia and abundant in rainforests; and **B** the typical rainforest habitat where they can be found (pictured: Washpool National Park, NSW, Australia). Photographs were taken by NB

Here, we perform extensive field collections throughout rainforest fragments in southeast Australia and use genotype-by-sequencing (GBS) through the DarTseq™ platform to obtain genetic information (single nucleotide polymorphisms; ‘SNPs’), targeting a species that is endemic to the region – *Chrysomya latifrons* Malloch 1927 (Fig. 1). This highly mobile species is frequently collected in fragmented rainforests across a large (~ 1000 km) geographic range. We expect that *Ch. latifrons* should show high levels of gene flow and limited genetic differentiation between adjacent rainforests. As we expect to observe only broad patterns of isolation by distance, any rainforest fragments inhabited by *Ch. latifrons* that do not meet these patterns may reflect high levels of habitat isolation.

## Methods

### Study species

*Ch. latifrons* has generally been considered a rainforest specialist that is restricted to New South Wales (NSW), Australia (Malloch 1927; Wallman 2002) and has been commonly recorded throughout rainforests ranging from Wollongong to Coffs Harbour (Butterworth et al. 2020). However, it has also been collected in rainforest-adjacent dry eucalypt forests (Dawson et al. 2021), as well as in urban green spaces around the Sydney region (Kavazos and Wallman 2012). As such, *Ch. latifrons* appear to be capable of tolerating a wide range of habitats but most commonly encountered in rainforests.

### Specimen collecting

To create a bait that was attractive to *Ch. latifrons*, 500 g of raw kangaroo mince was left outdoors for 72 h to attract *Calliphora* blowflies. Once *Calliphora* larvae had fed on the meat and reached the third instar, we tightly sealed the container for 24 h. The subsequent anaerobic decomposition of *Calliphora* maggots (in combination with the decomposing kangaroo mince) results in the production of specific volatiles that are highly attractive to all Australian *Chrysomya* species (except *Chrysomya flavifrons* Aldrich 1925). This bait presumably exploits the preference of *Chrysomya* species for cues associated with carrion that is in the mid-stage of decomposition, as most *Chrysomya* are secondary colonisers (Dawson et al. 2021).

**Fig. 2:**
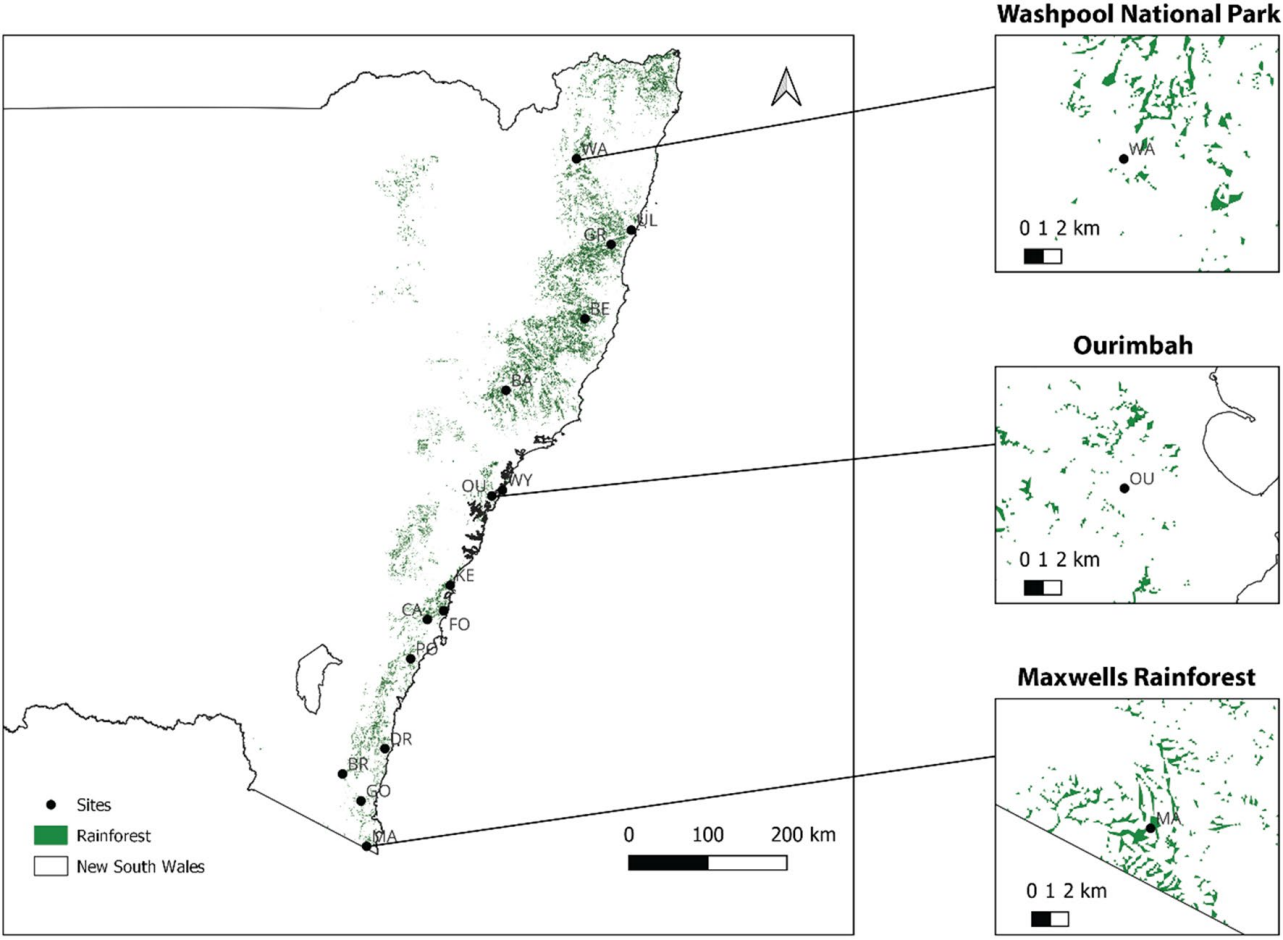
Spatial structure of rainforests (represented by dark green) throughout New South Wales, Australia. Data sourced from the Australian State of the Forests 2018 report (https://www.agriculture.gov.au/abares/forestsaustralia/sofr/sofr-2018). Rainforest habitat was defined as “Forest dominated by broad-leaved tree species, typically in wet or sheltered environments and with a closed canopy. Can include areas with non-rainforest species as emergents (trees emerging above the canopy), but where rainforest species dominate the character of the site”. Spatial fragmentation of rainforests at the local scale (10 km) is also represented for three populations (WA, OU, MA) in the right panels

Baits were used to collect a total of 188 *Ch. latifrons* adults with sweep nets at rainforest sites between Washpool, NSW and Maxwells Rainforest, NSW (for all rainforest sampling locations refer to Table 1, Fig. 2). Sampling trips were conducted between November 2020 and February 2021, and specimens were only collected at each site during a single round of sampling (between 2 to 6 h per site). The collection location at each rainforest was chosen at random and was not expected to bias signatures of genetic structure, as blowflies are strong dispersers and are finely tuned to the detection of carrion (Norris 1959), so any blowflies caught on the bait will broadly represent that rainforest’s genetic diversity. Flies were euthanised with ethyl acetate vapour within eight hours of capture, taxonomically identified following the key of Wallman (2002), placed into 2.5 mL plastic tubes containing 90% ethanol, and stored at − 4 °C in the laboratory for up to eight weeks.

**Table 1:** Locality information, collection dates, and sample numbers from each of the sites where *Chrysomya latifrons* (Diptera: Calliphoridae) was collected

Site	Abbr	Lat	Lon	Date	#	Ho	He	F_IS_	F_ST_
Barrington Tops National Park	BA	− 32.1508	151.5248	27/01/21	16	0.180	0.212	0.129	0.000
Bellangry	BE	− 31.2892	152.5370	01/02/21	12	0.173	0.210	0.139	0.009
Brown Mountain	BR	− 36.5972	149.4440	18/02/21	10	0.171	0.206	0.126	0.032
Foxground	FO	− 34.6982	150.7920	26/11/20	12	0.173	0.211	0.144	0.007
Goodenia Rainforest	GO	− 36.8990	149.7154	20/02/21	16	0.175	0.213	0.138	0.000
Grahams Trail	GR	− 30.4245	152.8304	28/01/21	14	0.174	0.211	0.150	0.004
Maxwells Rainforest	MA	− 37.4145	149.8138	19/02/21	18	0.173	0.212	0.150	0.000
Mt Cambewarra	CA	− 34.8049	150.5722	07/12/20	12	0.173	0.210	0.137	0.009
Mt Dromedary	DR	− 36.2946	150.0337	17/02/21	17	0.183	0.212	0.114	0.000
Mt Keira	KE	− 34.4047	150.8713	24/11/21	12	0.177	0.210	0.124	0.008
Ourimbah	OU	− 33.3649	151.3939	26/01/21	12	0.177	0.209	0.118	0.013
Pointer Gap	PO	− 35.2605	150.3582	13/12/20	12	0.175	0.212	0.140	0.001
Ulidarra National Park	UL	− 30.2488	153.0855	31/01/21	3	0.174	0.210	0.086	0.039
Washpool National Park	WA	− 29.4700	152.3160	29/01/21	16	0.180	0.211	0.120	0.002
Wyrrabalong National Park	WY	− 33.2938	151.5356	26/01/21	5	0.171	0.208	0.124	0.032

We also provide mean values of observed heterozygosity (Ho), expected heterozygosity (He), inbreeding coefficients (F_IS_) per population, and population-specific F_ST_ (Weir and Goudet 2017)

### Rainforest characteristics

To map the spatial distribution of rainforest fragments and to estimate levels of rainforest fragmentation, we downloaded the dataset from the Australian State of the Forests 2018 report (https://www.agriculture.gov.au/abares/forestsaustralia/sofr/sofr-2018), which characterises rainforests as “Forest dominated by broad-leaved tree species, typically in wet or sheltered environments and with a closed canopy. Can include areas with non-rainforest species as emergents (trees emerging above the canopy), but where rainforest species dominate the character of the site”. We then used QGIS version 3.28.1 (http://www.qgis.org/) to filter this dataset on the rainforest, exported this data from raster to polygons for mapping, clipped to the extents of NSW, and created a 10 km and 60 km buffer around each site. We chose both scales as they represent the range of observed dispersal capacities of *Chrysomya* blowflies (Norris 1959; Braack and Retief 1986). By incorporating both spatial scales, we can, therefore, see how the extent of patch connectivity and fragmentation change within the spatial extent that is relevant to the focal organism – where truly ‘isolated’ patches should show a low number of fragments and total area at both spatial scales. We intersected both the 10 km and 60 km buffers with the rainforest dataset to return the total number of rainforest patches and dissolved these resulting features to get estimates of the total rainforest area. Lastly, we divided the rainforest area by count to calculate the average size of patches based on the 10 km and 60 km buffers. Together, these characteristics provide proxies of rainforest fragmentation surrounding each collection locality – where fewer patches, less total rainforest area, and lower average patch sizes represent higher levels of fragmentation.

### DArTSeq™ assay

The head of each individual fly (3–18 from each population) was dissected from the body, placed in a single well of a 96-well plate with 70% ethanol, and supplied to Diversity Arrays Pty Ltd (Canberra, Australia) for a high-density DArTSeq™ assay (~ 2.5 million sequences/sample used in marker calling). The DArTSeq™ extraction and sequencing methods are detailed in Kilian et al. (2012) and Georges et al. (2018). In brief, restriction enzyme digestion is utilised to digest genomic DNA into fragments which are then sequenced using an Illumina short-read platform. Short-read sequences are then bioinformatically processed to produce a series of SNPs.

To ensure appropriate DNA fragments were used for subsequent sequencing, restriction enzyme digestion was optimised for *Ch. latifrons* using multiple restriction enzyme combinations and eight specimen replicates. Following sequencing of these test specimens, the optimal restriction enzyme pair was identified as PstI-HpaII, based on the fraction of the genome represented, while controlling for average read depth and the number of polymorphic loci. This restriction enzyme combination was used for all subsequent digestions. Following digestion, all sequence fragment libraries were ligated with Illumina sequencing adaptors and sequenced on an Illumina HiSeq2000 platform.

Short-read sequences were processed using the DarTseq™ bioinformatic pipeline (Georges et al. 2018), which performs filtering and variant calling, to generate final genotypes. While some parts of the sequencing and analysis protocol cannot be provided, the use of the DArTSeq platform for studies of genetic diversity and structure is widespread (Popa‑Báez et al. 2020; Hoffman et al. 2021; Jaya et al. 2022) and is reproducible because subsequent use of this proprietary service will yield the same results.

### SNP filtering

The DArTseq™ dataset contained a total of 21006 SNPs across 187 individual flies (Supplementary material 1), as a single specimen from Washpool National Park was removed from the dataset due to low quality reads. The data were then filtered with the ‘dartR’ package version 1.9.9.1 (Gruber et al. 2018) in R version 3.6.1 (R Core Team 2019). It is generally recommended that a range of data filtering thresholds are applied and tested for the same dataset (Wright et al. 2019; Schmidt et al. 2021), particularly because optimal metrics and thresholds will differ between analyses (Linck and Battey 2019; Hoffmann et al. 2021). As such, we first filtered the DArTseq™ dataset by reproducibility (proportion of technical replicate assay pairs for which the marker score was consistent) at a threshold of 0.98, then by call rate (proportion of samples for which the genotype call was not missing) at a threshold of 0.95, and finally by minor allele count (MAC less than the threshold are removed) at a threshold of 3. This resulted in a filtered dataset of 187 individual genotypes, 3910 SNPs, and 1.22% missing data. To compare the impact of MAC vs minor allele frequency (MAF) filtering, we next filtered the original dataset using the same parameters above but with a MAF threshold of 0.02 instead of a MAC of 3. This resulted in a final dataset of 187 individual genotypes, 2693 SNPs, and 1.25% missing data, which was used for all downstream analyses. As has been recommended for analyses of population structure (Linck and Battey 2019; Hoffmann et al. 2021), we used the MAC-filtered DArTseq™ dataset as a comparison for later admixture analyses, and the MAF-filtered dataset for all other downstream analyses (see results).

To consider the effect of assembly parameters on alignments and downstream analyses (e.g., Linck and Battey 2019), we also processed the raw fastq files from DArT-Seq™ samples with IPYRAD v. 0.7.28 (Eaton and Overcast 2020), to filter and remove low-quality data, identify homology among reads through de novo assembly, make SNP calls, and create a variant call format (VCF) file of variant sites (https://ipyrad.readthedocs.io/en/master/). Default settings were used in IPYRAD, with the exception of the following: strict filtering of adapters was applied (filter_adapters = 2; default 0—no filtering); and the final minimum length after filtering was set to 60 (filter_min_trim_len = 60; default 35). The IPYRAD dataset contained a total of 48,912 SNPs across 187 individual flies. The data were then filtered in VCFTOOLS v. 0.1.13 (Danecek et al. 2011) using –missing-indv, –max-missing-count, and –maf filters to remove any individuals with more than 50% missing data, any sites with more than 20% missing genotypes, and any sites with a MAF of less than 5%, respectively. This resulted in a final dataset of 7862 SNPs.

### Genetic diversity

We used R for all analyses of genetic diversity, and unless otherwise stated, all analyses were conducted using the DArTseq™ genotype dataset filtered for a MAF of 0.02. We first applied the ‘basic.stats’ function of the ‘hierfstat’ package version 0.5–10 (Goudet et al. 2015) to calculate average observed heterozygosity (H_O_), expected heterozygosity (H_E_), and inbreeding coefficients (F_IS_). We also used the ‘betas’ function from ‘hierfstat’ to calculate population-specific F_ST_ values (Weir and Goudet 2017).

### Population structure

Using R, we first assessed population structure by AMOVA using the function ‘poppr.amova’ with the ‘ade4’ implementation from the ‘poppr’ package version 2.9.3 (Kamvar et al. 2014). To test whether populations were significantly different, we used a randomisation test on the AMOVA output with 1000 permutations (Excoffier et al. 1992) using the function ‘randtest’ from the package ‘ade4’ version 1.7–18 (Thioulouse et al. 2018). We then conducted pairwise comparisons of F_ST_ values between populations using the ‘gl.fst.pop’ function from the ‘dartR’ package with 10,000 bootstrap replicates.

Genetic distances between individuals were examined using Nei’s distances, and a dendrogram with 1000 bootstrap replicates was created with the ‘aboot’ function of the ‘poppr’ package, and the ‘ggtree’ function of the package ‘ggtree’ (Yu 2020). We then used the ‘glPca’ function from the ‘adegenet’ package version 2.1.5 (Jombart 2008) to determine whether genetic differences between individuals (as represented by principal components) were structured according to their populations.

To test for isolation by distance, we performed a Mantel test using the function ‘gl.ibd’ from the ‘dartR’ package in R. This compared linearised genetic distances between populations (calculated using ‘StAMPP’ version 1.6.3; Pembleton et al. 2013) against Euclidean geographical distances (calculated using ‘vegan’ version 2.5–7; Oksanen et al. 2013). We followed this with a mantel correlogram analysis using the ‘vegan’ function ‘mantel.correlog’.

To calculate individual blowfly admixture coefficients, the MAF-filtered SNP data were converted into the STRUCTURE format (‘.str’) using the ‘gl2faststructure’ function from the ‘dartR’ package, then into the ‘.geno’ format using the ‘struct2geno’ function of the ‘LEA’ package version 3.1.4 (Frichot and Francois 2015). We then ran sparse non-negative matrix factorisation on these data with the ‘sNMF’ function from ‘LEA’. We analysed *K* values of 1 to 10, with 100 replications for each *K* value, and used the cross-entropy criterion to determine the value of *K* that best explained the results. To assess whether filtering had a major impact on signatures of population structure, we ran this analysis a second time on the DArTseq™ dataset after filtering for a MAC of 3, as has been recommended for analyses of population structure (see above).

To verify these results across our different bioinformatic pipelines, we also analysed the IPYRAD-filtered dataset using sNMF (with the same parameters as above) and tested the IPYRAD-filtered dataset with the program STRUCTURE v.2.3.4 (https://web.stanford.edu/group/pritchardlab/structure.html) across a *K* value range of 1 to 10 with 10 replications per *K*. An initial analysis was completed to determine the number of Markov Chain Monte Carlo (MCMC) iterations required to achieve stationarity in F_ST_ and log(α) summary statistics for each *K* value. The optimal configuration was determined to be 100,000 MCMC iterations, with 5000 discarded as burn in and these parameters were used for all subsequent analyses. Following STRUCTURE analysis, the best *K* value was selected using the Evanno method (Evanno et al. 2005) implemented within the Structure Harvester online interface (available at: http://taylor0.biology.ucla.edu/structureHarvester/). To create plots, results for each *K* value were permuted across all replicates using CLUMPP v1.1.2 (Jakobsson and Rosenberg 2007) and then plotted using Toyplot 0.18.0 (https://github.com/sandialabs/toyplot).

### Gene flow

Finally, to calculate relative migration rates among populations, we used the divMigrate implementation (Sundqvist et al. 2016) of the ‘diveRsity’ package version 1.9.90 (Keenan et al. 2013) in R. The directional migration calculations implemented by divMigrate require that populations are predetermined (Sundqvist et al. 2016), however, we were unable to make any population assignments based on the sNMF or STRUCTURE analyses. As such, we performed two separate analyses: the first with all 15 geographic populations, and the second with individuals only from the northern-most population (WA), the central population (KE), and the southern-most population (MA) – on the assumption that these are the most geographically isolated and should show the lowest levels of directional gene-flow. For both analyses, we estimated 50 bootstrap iterations, set the filter threshold to 0.2, and chose the effective number of migrants (*Nm*) to infer levels of connectivity between populations.

## Results

Rainforest fragmentation is extensive at both local scales of 10 km and broader scales of 60 km, with often hundreds of small rainforest patches spread throughout drier sclerophyll forests. Fragmentation is particularly pronounced at lower latitudes (Fig. 2; Table 2). We see that Wyrrabalong National Park (WY) which is composed of one major rainforest patch and appears relatively isolated at a scale of 10 km is in fact relatively well connected at a scale of 60 km (which is within the dispersal kernel of *Ch. latifrons*). Alternatively, Maxwell’s Rainforest (MA) which appears relatively well connected at a scale of 10 km is in fact quite isolated at a scale of 60 km – and would thus be expected to have the lowest level of broad connectivity to other patches. Nevertheless, at most sites, *Ch. latifrons* were abundant at the time of collection, ranging from a constant number of five to 20 individuals on the carrion bait. However, at Ulidarra National Park (UL) we only observed three *Ch. latifrons* as the community was dominated by *Ch. semimetallica* – suggesting this may be close to the range edge of *Ch. latifrons*. At Wyrrabalong National Park (WY) we only observed five flies throughout four hours of collecting (Table 2).

**Table 2:** Spatial characteristics of the rainforest fragments where *Chrysomya latifrons* (Diptera: Calliphoridae) was collected

Site	Total hectares (10 km)	Total hectares (60 km)	Patch # (10 km)	Patch # (60 km)	Avg patch size (10 km)	Avg patch size (60 km)
BA	1309	33,628	541	9644	2.42	3.49
BE	4036^a^	48,648^a^	932^a^	11,451^a^	4.33^a^	4.25^a^
BR	440	5826	177	2797	2.49	2.08
FO	1602	10,185	784	4177	2.04	2.44
GO	329	5166	182	2584	1.81	1.99*
GR	1616	38,276	676	10,787	2.39	3.55
MA	992	2596*	391	1192*	2.54	2.18
CA	1222	11,820	625	4660	1.96	2.54
DR	975	9694	371	4178	2.63	2.32
KE	953	9011	440	4062	2.17	2.22
OU	627	4384	322	2051	1.95	2.14
PO	1186	9357	355	3438	3.34	2.72
UL	898	23,221	334	7837	2.69	2.96
WA	1150	14,901	309	4459	3.72	3.34
WY	1.36*	4251	1*	2011	1.36*	2.11

Total hectares is the sum of the area of all rainforest polygons within 10 km or 60 km of the collection coordinates

Patch number is the number of unique rainforest polygons within 10 km or 60 km of the collection coordinates. Average patch size is the total hectares divided by the number of patches. Within each column, the largest and smallest values are shaded in grey where

*Represents the smallest value

^a^Represents the largest value

### Genetic diversity

Unless otherwise stated, all results relate to the DArTseq™ dataset filtered for a MAF of 0.02. Across all SNP loci, 75% of individuals were homozygous for the reference allele, 7% were homozygous for the alternate allele, and 18% were heterozygous – indicating relatively low genetic diversity across all populations. Low heterozygosity was also observed within each population, with population-specific H_O_ values all < 0.180, and all lower than the average expected heterozygosity (H_E_) (Table 1). Further to this, more than 30% of loci had an average H_O_ of ≤ 0.10, and only 20% of loci had an average H_O_ > 0.30 (Supplementary material 2: Fig. 1). Despite H_O_ being less than H_E_ in all populations, no strong signatures of inbreeding were detected, with low F_IS_ values in each population (always < 0.150; Table 1).

### Population structure

Population-specific F_ST_ values were low (ranging from 0.000 to 0.032), suggesting minimal genetic differentiation between populations. Likewise, pairwise comparisons of F_ST_ values between populations were all < 0.009, and there were no significant differences detectable between geographic populations (Table 3). AMOVA indicated that most of the genetic variation was observed within individuals (83.5%) followed by between individuals (16.5%), and both were found to be significant factors contributing to the overall population structure (*P* < 0.01) (Table 4). However, genetic variation between populations was not detected (0.00%), consistent with the F_ST_ results.

**Table 3:** Pairwise F_ST_ values between 15 populations of *Chrysomya latifrons* (Diptera: Calliphoridae). Population abbreviations are provided in Table 1

	KE	FO	CA	PO	BA	UL	BE	OU	WY	GR	WA	DR	BR	MA	GO
KE	–	–	–	–											
FO	0.000	–	–	–	–	–	–	–	–	–	–	–	–	–	–
CA	0.000	0.002	–	–	–	–	–	–	–	–	–	–	–	–	–
PO	0.000	0.000	0.000	–	–	–	–	–	–	–	–	–	–	–	–
BA	0.000	0.000	0.000	0.001	–	–	–	–	–	–	–	–	–	–	–
UL	0.000	0.000	0.003	0.000	0.000	–	–	–	–	–	–	–	–	–	–
BE	0.000	0.000	0.001	0.000	0.000	0.000	–	–	–	–	–	–	–	–	–
OU	0.000	0.000	0.001	0.000	0.000	0.000	0.000	–	–	–	–	–	–	–	–
WY	0.002	0.002	0.008	0.001	0.000	0.000	0.000	0.003	–	–	–	–	–	–	–
GR	0.000	0.000	0.000	0.000	0.000	0.000	0.000	0.000	0.003	–	–	–	–	–	–
WA	0.000	0.000	0.001	0.002	0.000	0.000	0.000	0.000	0.005	0.000	–	–	–	–	–
DR	0.000	0.000	0.000	0.000	0.000	0.004	0.000	0.000	0.003	0.000	0.001	–	–	–	–
BR	0.001	0.000	0.001	0.000	0.001	0.005	0.000	0.003	0.005	0.000	0.000	0.001	–	–	–
MA	0.001	0.000	0.001	0.000	0.000	0.000	0.001	0.000	0.002	0.000	0.001	0.000	0.002	–	–
GO	0.000	0.000	0.000	0.000	0.000	0.000	0.000	0.000	0.002	0.000	0.002	0.001	0.001	0.000	–

**Table 4:** Analysis of molecular variance (AMOVA) to assess the extent of variation within and between 15 populations of *Chrysomya latifrons* (Diptera: Calliphoridae)

Source	df	SS	MS	Est. Var	PV (%)	*P*-Value
Between populations	14	9225.6	658.9	0.0	0.0	0.601
Between individuals	172	11,3670.2	660.9	94.4	16.5	0.001*
Within individuals	187	88,596.2	473.8	473.8	83.5	0.001*
Total	373	21,1491.9	567.0	567.2	100	**–**

*df* degrees of freedom, *SS* sum of squares, *MS* mean square, *Est. Var* estimated variance *PV* percentage variance

**Fig. 3:**
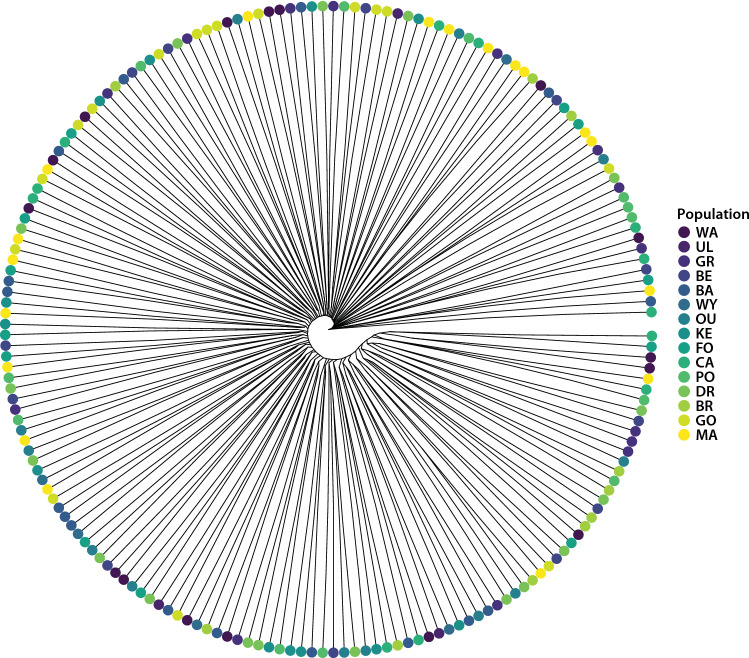
A dendrogram based on Nei’s genetic distances for individual *Chrysomya latifrons* (Diptera: Calliphoridae) from 15 rainforest populations. Populations are coloured according to the provided key in order from north (dark purple) to south (yellow). Population abbreviations are provided in Table 1

**Fig. 4:**
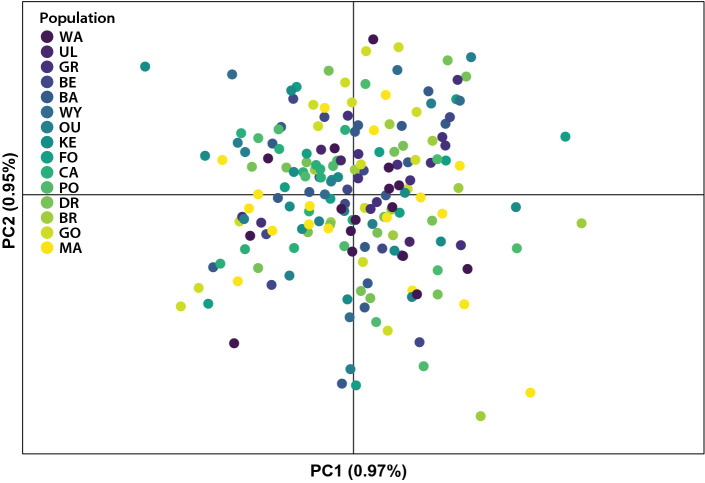
Principal component analysis of the filtered dataset of 2693 SNP loci from 15 populations of *Chrysomya latifrons* (Diptera: Calliphoridae). Populations are coloured according to the provided key in order from north (dark purple) to the south (yellow). Population abbreviations are provided in Table 1

There was also no clear differentiation between individuals from different populations based on Nei’s genetic distances. In fact, individuals from populations separated by > 900 km often showed greater genetic similarity to each other than to individuals that were collected from the same geographic population (Fig. 3). Consistent with this, both the Mantel test of genetic versus geographic distance and the Mantel correlogram yielded no significant correlations (Mantel’s *r* = − 0.053, *P* = 0.685) (Supplementary material 2: Fig. 2a, b). Likewise, in the principal component analysis (Fig. 4, Supplementary material 2: Fig. 3), the first two principal components explained only 0.97% and 0.95% of the total variation, and there was no clear separation of populations based on individual principal component scores (Fig. 3).

**Fig. 5:**
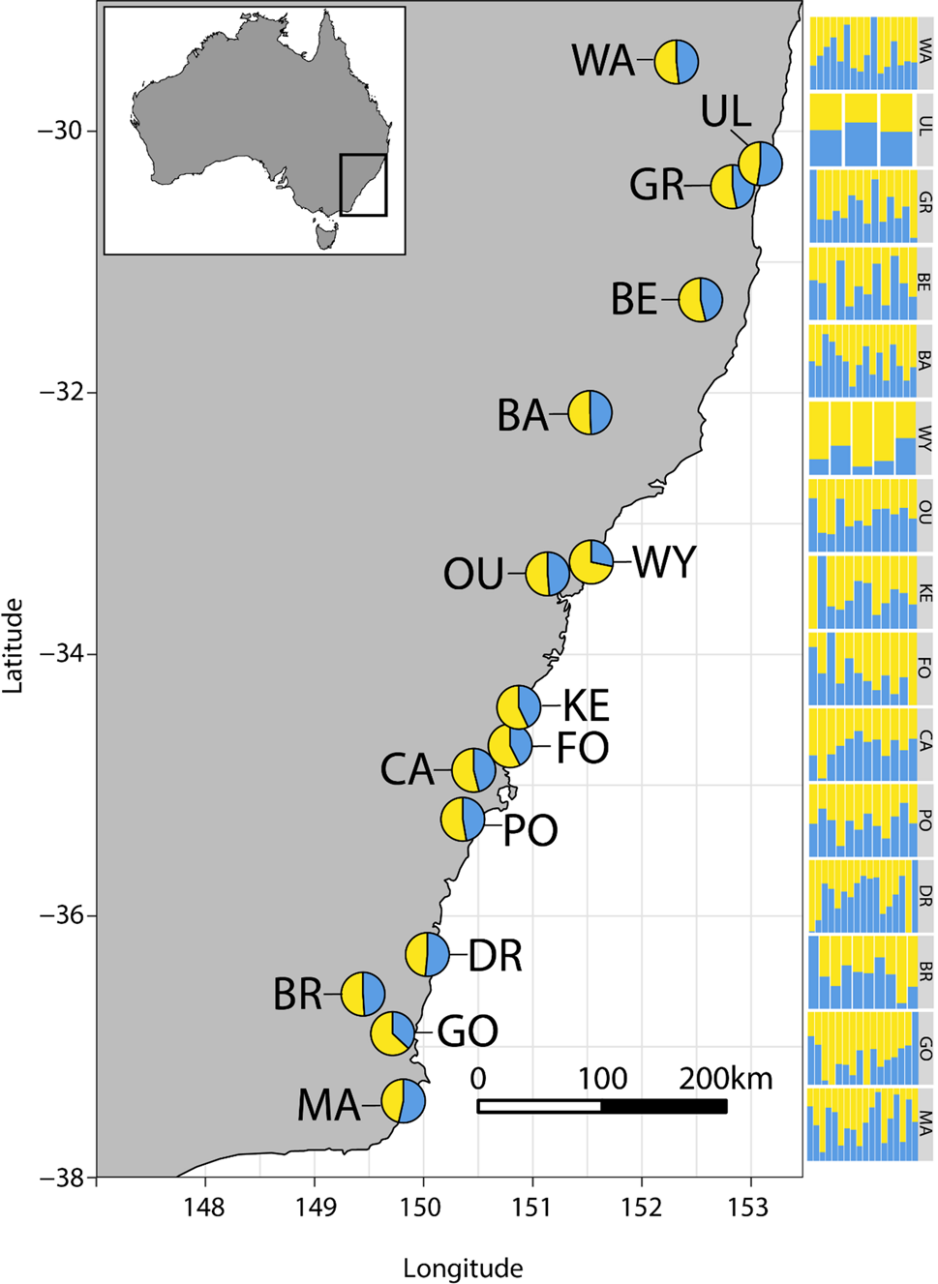
The mean admixture proportions of populations of *Chrysomya latifrons* (Diptera: Calliphoridae) that were sampled in the present study. The admixture proportions plotted on the map represent population averages. The bar plots presented on the right reflect individual admixture proportions, sorted by population, where each bar represents a single individual. Full population names are provided in Table 1. All analyses and plotting can be replicated by following the online tutorial provided by Tom Jenkins (https://github.com/Tom-Jenkins/admixture_pie_chart_map_tutorial)

The sNMF analyses of all three datasets (MAF = 0.02, MAC = 3, and IPYRAD) all identified the lowest cross-entropy for a *K* value of one (Supplementary material 2: Fig. 4), and all showed similar admixture proportions (Supplementary material 2: Fig. 5) suggesting that all geographic populations share a common ancestry. To investigate admixture in more detail, we explored the results from the MAF = 0.02 filtered dataset for *K*-values from 1 to 10. For all tested *K*-values > 1, we found that admixture was evenly spread throughout geographic locations – supporting the notion that all populations share a common ancestry and that none were substantially genetically differentiated. To visualise these admixture proportions, we present the results for *K* = 2 in Fig. 5.

**Fig. 6:**
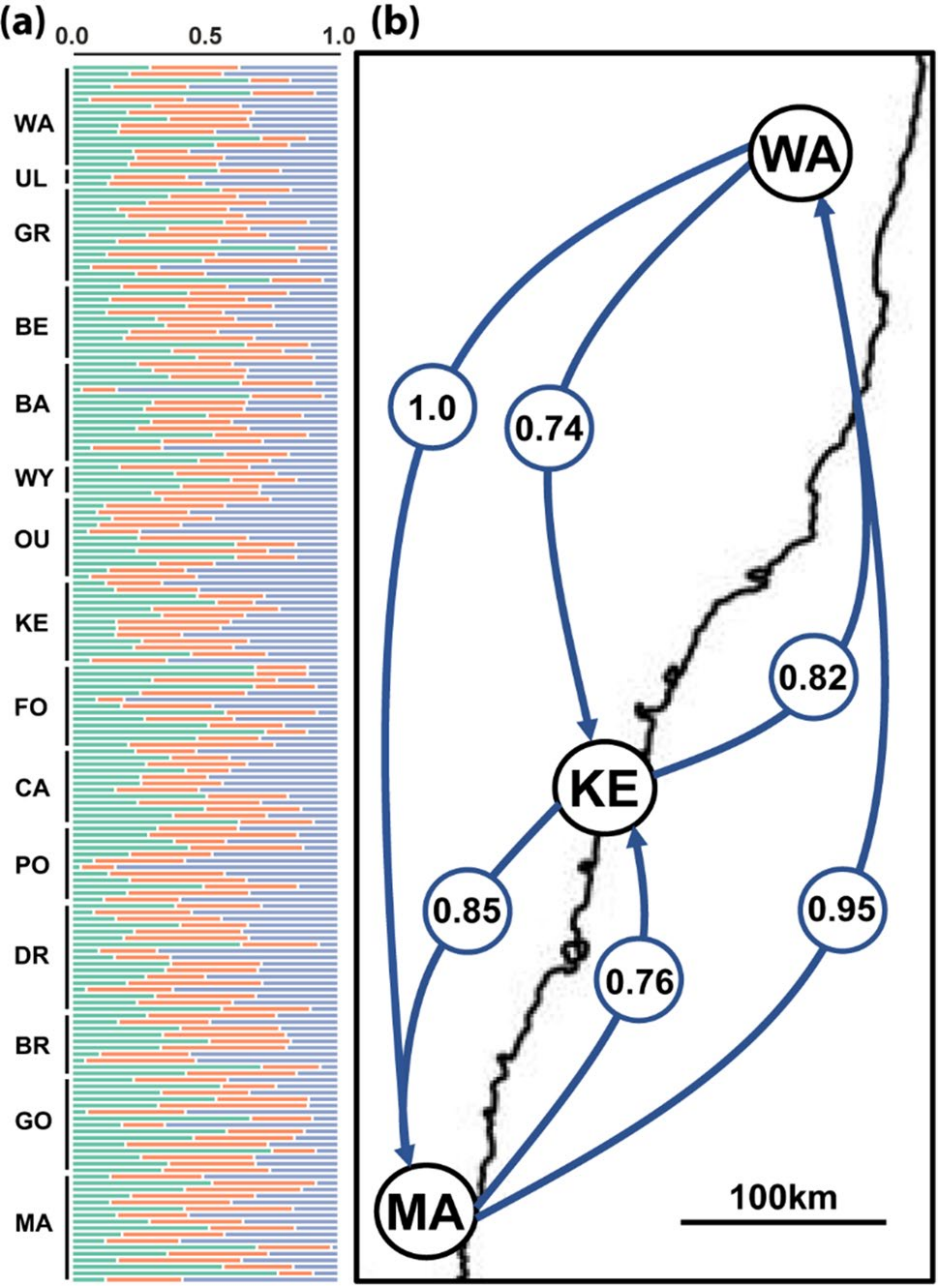
Geographic distribution of genetic structure and gene flow in *Chrysomya latifrons*. Full population names are provided in Table 1. **a** STRUCTURE plot of genetic assignment (*K* = 3) for 187 individuals. Populations are sorted from top to bottom geographically (north to south). **b** A relative migration network. Letters represent sampling sites, arrows mark the direction of gene flow, and numbers represent the level of migrant exchange between locations (i.e., effective number of migrants *Nm*) (Sundqvist et al. 2016). Results of the same analysis with all 15 geographic populations are presented in Supplementary material 2: Table 1

Likewise, the STRUCTURE analysis based on the IPYRAD dataset (Fig. 6a) was concordant with the sNMF analyses and suggested no clear genetic differentiation between populations at *K* = 3 (the optimal *K* value supported by the Evanno method).

### Gene flow

Gene flow was generally high among all 15 populations (Supplementary material 2: Table 1), with numbers of effective migrants (*Nm*) > 0.5 with the exception of the WY and UL populations (which both had low sample sizes of *N* ≤ 5), and with no significant asymmetries in migration rates between population pairs. When considering only the three most isolated populations (WA, KE, and MA), gene flow was again determined to be high in all cases (*Nm* ≥ 0.74) with no significant asymmetries in migration rates between population pairs (Fig. 6b).

## Discussion

We assessed genetic connectivity among fragmented rainforests in the endemic Australian blowfly *Ch. latifrons*. Based on the ubiquity, widespread distribution, and high dispersal ability of *Ch. latifrons*, we expected to find limited genetic differences between adjacent rainforests and only a broad pattern of isolation by distance. However, we found a complete lack of genetic structure between populations – indicative of a single large, panmictic population despite the ~ 1000 km range distribution span. Broadly, this highlights that strongly dispersing insects, such as blowflies, may have the capacity to disperse between and connect isolated rainforest fragments.

### Distribution and biology

We found *Ch. latifrons* in high abundances (at the time of collection) from Washpool National Park in northern NSW to Maxwells Rainforest on the southern border with Victoria. This is the first comprehensive information on the distribution of *Ch. latifrons*, which inhabits a large latitudinal transect of > 1000 km of eastern Australian rainforests and rainforest-adjacent sclerophyll forests. It is almost certain that populations also extend into the rainforests of northern Victoria, where there is suitable habitat available. Remarkably however, *Ch. latifrons* was completely absent near the Queensland border, where its closest genetic relative, *Ch. semimetallica*, occurs (Butterworth et al. 2020). These two species are rarely collected at the same location and likely compete with each other in a thin hybrid zone between Coffs Harbour in NSW and Brisbane in Queensland – providing an interesting model system for future work focused on understanding the eco-evolutionary dynamics of allopatry.

It was clear from our sampling that *Ch. latifrons* inhabits every major rainforest in south-eastern Australia, often greatly outnumbering other carrion-breeding flies on a given resource during the days we sampled – it was not uncommon to see 30–40 individuals around larger resources in the mid-stages of decomposition. Importantly, however, our observations of abundance are based only on one-time point. Although consistent with high numbers observed in previous studies (Kavazos and Wallman 2012; Butterworth et al. 2020; Dawson et al. 2021), there is likely to be significant year-to-year variation in abundances as well as temporal variability throughout the day. Without more extensive and repeated sampling, we are unable to make any general conclusions on the abundance of *Ch. latifrons*. In fact, this is the case for most rainforest invertebrates, particularly decomposers. There is still much to learn about seasonal and temporal drivers of the distribution and abundance of invertebrates in Australia; such knowledge will be instrumental to understanding the functioning of Australian ecosystems.

Considering the widespread distribution of *Ch. latifrons* and its common occurrence in rainforest carrion, it is likely playing an important role in rainforest ecosystems as a primary decomposer, but its biology is poorly understood. It is likely to be a carrion-breeding species, infesting vertebrate carrion in rainforests and adjacent habitats. This is supported by its attraction to carrion baits, oviposition on carrion, and the similarity of its larvae to other closely related carrion-breeding generalist species (Szpila and Wallman 2016). The widespread distribution and ability of *Ch. latifrons* to easily disperse long distances between habitats (see below) suggests they are either generalist consumers of a wide range of animal carrion types, or specialist consumers that utilise types of carrion that are abundant throughout rainforests.

### Genetic diversity and population structure

Values of observed heterozygosity < 0.30 are considered to represent low genetic diversity across a wide range of animals (Andere et al. 2008, 2020; Robertson et al. 2018; Kleinhans and Willows-Munro 2019; Melo-Carrillo et al. 2020). For all the populations sampled in the present study, the mean observed heterozygosity was < 0.18. In fact, more than 30% of loci had an observed heterozygosity of ≤ 0.10, and observed heterozygosity was consistently lower than expected heterozygosity. These values of observed heterozygosity are particularly low when compared to many other strongly-dispersing fly species, where average levels of observed heterozygosity in microsatellite markers between 0.3 and 0.7 have been recorded (Wilke et al. 2017; Qin et al. 2018; Bateta et al. 2020; Deschepper et al. 2021). Low levels of heterozygosity seem unlikely to be due solely to inbreeding, as signatures of inbreeding were not strong based on F_IS_. Rather, they may simply be the result of significant historical bottlenecks (Menken 1987; Brouwer et al. 2007) or selection against heterozygotes (underdominance) (Láruson and Reed 2016). Broadly, this suggests a widespread lack of genetic diversity for *Ch. latifrons*, despite these flies being readily abundant during sampling, and spread over such a wide geographic range. In addition, population-specific F_ST_ values indicate that population structure in *Ch. latifrons* is extremely low or absent, and this was reinforced by the consistent results of AMOVA, Nei’s genetic distances, principal component analysis, isolation-by-distance, admixture, and STRUCTURE analyses. Taken together, the diversity and differentiation results suggest that *Ch. latifrons* exist within east Australian rainforests as one large panmictic and genetically depauperate population.

The low genetic diversity and lack of population structure observed in *Ch. latifrons* highlights that widely dispersing and abundant insects can nevertheless be genetically depauperate. Although it is expected that species competing on ephemeral resources should maintain high genetic diversity (Dytham and Shorrocks 1992), hypotheses regarding resource dynamics and population structure have rarely been tested in invertebrate populations. The unpredictable nature of the larval resources (which can also be adult mating sites) may lead to sweepstakes reproduction where population sizes are large due to high fecundity, but only a small number of individuals are able to successfully reproduce. Similar patterns of limited genetic diversity due to sweepstakes reproduction have been shown in other insects (Ruiz-Carbavo 2022).

Genetic diversity is expected to be closely linked to the adaptive capacity of populations (Hoffmann et al. 2017), so even though *Ch. latifrons* is widespread, it may be threatened by the drastic climatic and environmental changes (e.g., increasing fires and temperature; Legge et al. 2021) that are occurring along its habitat range. Particularly if these exert significant pressure on its limited pool of genetic diversity. A direct link between low genetic diversity and low adaptive potential has not been confirmed for most terrestrial invertebrates (Kellerman and van Heerwaarden 2019). However*,* the Australian fly species *Drosophila birchii* inhabits a similar stretch of rainforest fragments and, despite being widespread, has been shown to have limited additive genetic variance for desiccation resistance (Kellerman et al. 2006). Future studies should aim to characterise the adaptive capacity of *Ch. latifrons* (and a wider range of rainforest invertebrates), particularly in the context of tolerance to climate change – as rainforests continue to experience its increasingly severe and frequent effects (Jiang et al. 2019; Boulton et al. 2022). Such research should not only focus on rare or endangered species but also on common and widely distributed species that may play important roles in maintaining habitat connectivity between fragments.

### Potential explanations for lack of diversity and population structure

The low population structure we observed in *Ch. latifrons* is unlikely to solely be a result of their strong dispersal ability, as there is ample evidence that flying insects with strong dispersal capacities often still show high levels of population structure (e.g., Bateta et al. 2020; Bluher et al. 2020). One possible explanation may relate to migration patterns. *Chrysomya* species tend to thrive in warmer temperatures (Byrd and Butler 1997; Sontigun et al. 2018), so it is plausible that a major source population is maintained in the warmer northern rainforests during winter, from which numerous individuals may migrate towards southern rainforests over spring and summer.

This phenomenon is suggested to occur in Australian *Chrysomya rufifacies* (Norris 1959) as well as American populations of the blowfly *Cochliomyia hominivorax* (Eddy and Bushland 1956). Blowflies have been shown to disperse as far as 6 km within 24 h (Norris 1959), while some *Chrysomya* species can travel up to 65 km (Braack and Retief 1986), and individual stable flies (*Stomoxys calcitrans*) can migrate as far as 225 km (Hogsette and Ruff 1985). Thus, it is possible that long-distance migration from a small winter source population explains the lack of population structure of *Ch. latifrons*. In fact, many Australian insects are known to migrate great distances and change distributions between seasons. For example, *Mythimna* and *Helicoverpa* moths (Lepidoptera) both make long south-easterly migrations in the warmer spring and summer months (Drake and Gatehouse 1995; Satterfield et al. 2020).

Strongly dispersing species with long-distance seasonal migration patterns generally tend to show low population structure over broad geographic ranges (Endersby et al. 2007; Pfeiler and Markow 2017; Wang et al. 2021), further supporting the possibility that large numbers of *Ch. latifrons* undertake long migrations during the spring and summer. There are numerous rainforest pockets and corridors that could facilitate such migrations, particularly along the mountainous Great Dividing Range, which extends from Queensland to Victoria. However, our understanding of insect migration is still limited (Chapman et al. 2015; Pfeiler and Markow 2017; Satterfield et al. 2020), particularly as widespread distributions of insect populations in the summer months may mask the risk of local extinction of winter populations. This could occur in *Ch. latifrons*, particularly if a major source population is maintained in only a few northern NSW rainforests during the winter.

The spatial structure of carrion resources may also play a role, as these are often used as mating sites by blowflies (Butterworth et al. 2018, 2019). Carrion patches may thus act as ecological islands (Barton et al. 2013) between fragmented rainforests, facilitating genetic connectivity between patches at both local and broader scales. Physiological aspects may also play a role in driving population structure in *Ch. latifrons*. Some *Chrysomya* species can cope with temperatures as low as 11 °C (Cammack and Nelder 2010), and *Ch. latifrons* can occur around the greater Sydney region during the winter (Kavazos and Wallman 2012; Dawson et al. 2021). Individuals may diapause in southern regions throughout the winter (Saunders and Hayward 1998; Vinogradova and Reznik 2013), although there is no current evidence that *Ch. latifrons* has this capacity. Alternatively, the temperature and climatic differences between northern and southern rainforests during winter may not be drastic enough to prevent *Ch. latifrons* from inhabiting southern rainforests throughout the entire year. In this case, panmixia may be the result of more continuous patterns of breeding between rainforests along eastern Australia. A lack of genetic diversity and population structure has also been shown along a north–south transect of Queensland fruit fly populations (Popa‑Báez et al. 2020), supporting the idea that temperature variation along south-eastern Australia may not markedly constrain fly movement.

Finally, the lack of population structure in *Ch. latifrons* may not reflect the current population dynamics of the species. In the case of recent fragmentation (due to deforestation or wildfires) and large enough population sizes, there may not have been sufficient time for a resulting lack of gene flow to be reflected in patterns of population structure and diversity (i.e., Lloyd et al. 2013; Su et al. 2018; Delnevo et al. 2021). However, this seems unlikely, as *Ch. latifrons* can be found in habitats adjacent to rainforests (i.e., dry schlerophyll forests and urban areas) and almost certainly has the capacity to disperse through rainforest-adjacent habitats. Importantly, the lack of population structure we observed does not necessarily imply that there are no signatures of local adaptation in *Ch. latifrons*. Such questions could be addressed by measuring phenotypic differences between populations or with other sequencing approaches (e.g., genome skimming, ultra-conserved elements, exome, transcriptome) to investigate fine-scale signatures of local adaptation.

### Implications for rainforest connectivity

Our results provide important insights into broad patterns of rainforest connectivity in Australia – highlighting that rainforest inhabitants with strong dispersal capacities (such as blowflies) can likely move between, and connect highly fragmented habitats. Such widespread dispersal between rainforests is unlikely to be representative of all strong dispersers, as it depends largely on species-specific niche breadths and resource distributions that may constrain certain species to specific rainforest patches (e.g., Woltmann et al. 2012).

However, the ubiquitous distribution and movement of *Ch. latifrons* between isolated rainforests makes it a likely vector of dispersal for pollen, pathogens, parasites, and phoronts – forming an interconnected ecological network throughout southeast Australian rainforests. As such, we may also expect to see high degrees of genetic connectivity between the rainforest taxa that are associated with *Ch. latifrons*. Such correlated patterns of dispersal between fragmented habitats have been shown for rainforest trees and the African honeybees that pollinate them (Dick et al. 2003). Future work should, therefore, target the ecology of *Ch. latifrons*, particularly to understand their broad contributions to rainforest ecosystems and their adaptive potential in a changing climate.

### Conclusions

The future capacity of species to disperse between rainforests is likely to be hampered by ongoing climate change and deforestation, particularly with the increased likelihood of climatic disturbances (i.e., wildfires: Legge et al. 2021). Within Australia, the long-term effects of the devastating 2019–2020 bush fires on habitat fragmentation and population dynamics are yet to be fully seen, and the data we present for *Ch. latifrons* is a crucial reference point for long-term studies aimed at understanding these dynamics. There is, however, an urgent need to study the genetic structure and adaptive capacity of more poorly dispersing rainforest insects that are endemic to fragmented rainforests (Kellerman and van Heerwarden 2019). These insects are likely to be highly adapted to their specific habitats and are perhaps at the highest risk of local extinction due to climate change. Potential candidates are millipedes, worms, snails, ticks, spiders, and springtails – all of which are widely dispersed through rainforests (Mesibov 1998; Olson 1994) but lacking information on population genetic structure and adaptive capacity. We strongly encourage researchers to focus on such target species to further understand rainforest population dynamics and conservation priorities.

## Supplementary Information

Below is the link to the electronic supplementary material.

Supplementary file1 (CSV 26851 KB)

Supplementary file2 (DOCX 299 KB)

## Data Availability

Individual genotype data (SNP 1-row format) and sample metadata are available as supplementary data.

## Code availability

Available upon request.
