# Supplementary material for: The blowfly Chrysomya latifrons inhabits fragmented rainforests, but shows no population structure
Source: Oecologia. 2023 Feb 11;201(3):703–19. doi: 10.1007/s00442-023-05333-w (PMC10038970; doi:10.1007/s00442-023-05333-w)
Supplement: Supplementary file 2 — Supplementary file2 (DOCX 299 KB) [file 442_2023_5333_MOESM2_ESM.docx]

**Supplementary Material**

The blowfly *Chrysomya latifrons* inhabits fragmented rainforests, but shows no population structure

Nathan J. Butterworth (nathan.butterworth@monash.edu)^1,2*^, James F. Wallman ^2^, Nikolas P. Johnston^2,3,4^, Blake M. Dawson^4^, Joshua Sharp-Heward^4^, Angela McGaughran^5^

^1^School of Biological Sciences, Monash University, Clayton VIC 3800, Australia

^2^Faculty of Science, University of Technology Sydney, Ultimo NSW 2007, Australia

^3^Department of Ecology and Biogeography, Faculty of Biological and Veterinary Sciences, Nicolaus Copernicus University in Toruń, 87-100 Toruń, Poland

^4^Centre for Sustainable Ecosystem Solutions, School of Earth, Atmospheric and Life Sciences, University of Wollongong, Wollongong NSW 2522, Australia

^5^Te Aka Mātuatua - School of Science, University of Waikato, Private Bag 3105, Hamilton 3240, New Zealand

Corresponding author: nathan.butterworth@uts.edu.au


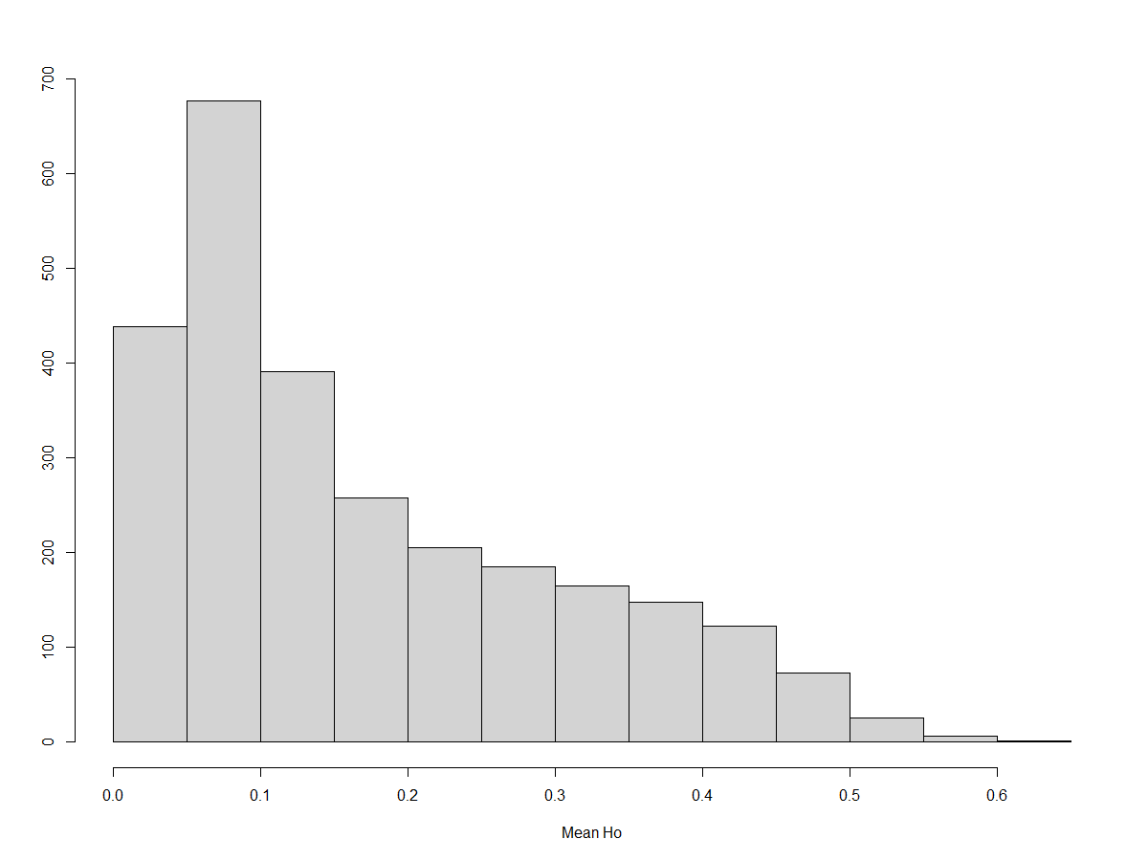


**Figure 1.** Mean observed heterozygosity (Ho) across the 2963 filtered SNP loci. Only 561 SNP loci (20.8%) had an average Ho greater than 0.3.


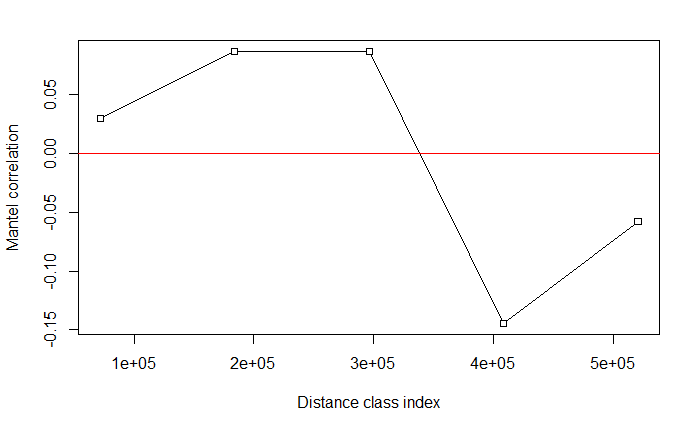

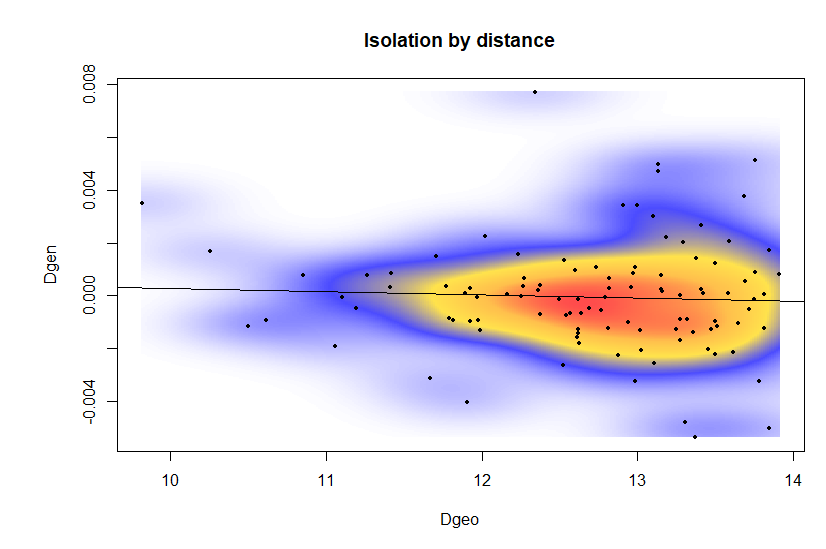


**(a)**

**(b)**

Geographic distance

Genetic distance

**Figure 2.** (a) Isolation by distance with the ‘gl.ibd’ function of the R package ‘dartR’ with 1000 permutations (r: -0.053, *P* = 0.685). Geographic distance represents the Euclidean distances among the latitude and longitude coordinates of each collection site, calculated using the ‘gl.ibd’ implementation in R (b) Mantel correlogram with the ‘mantel.correlog’ function of the R package ‘vegan’ with 999 permutations, non-significance represented by open white boxes. The distance class index represents the Haversine distances among the latitude and longitude coordinates of each collection site, calculated using the ‘distm’ function of the R package ‘geosphere’.


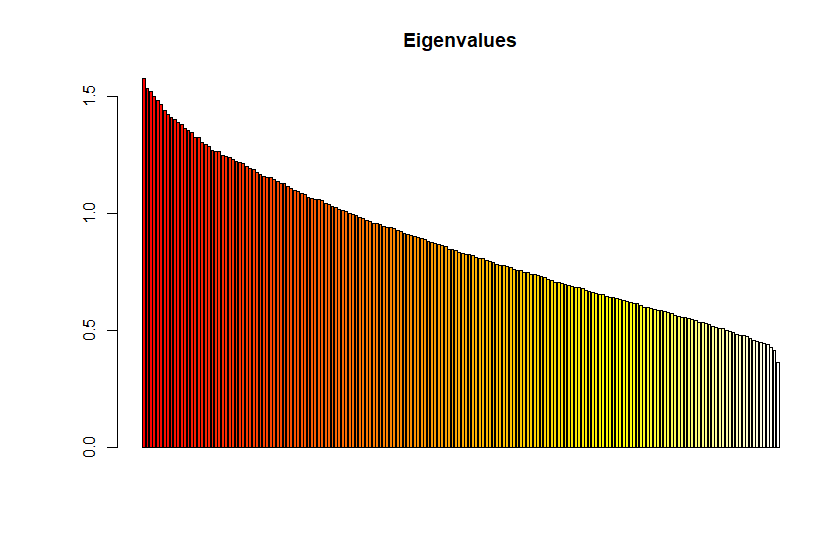


**Eigenvalues**

**Principal components (1 –> 186)**

**Figure 3.** Eigenvalues which represent the amount of variation explained by each principal component, attained from the ‘glPca’ function of the R package ‘adegenet’.


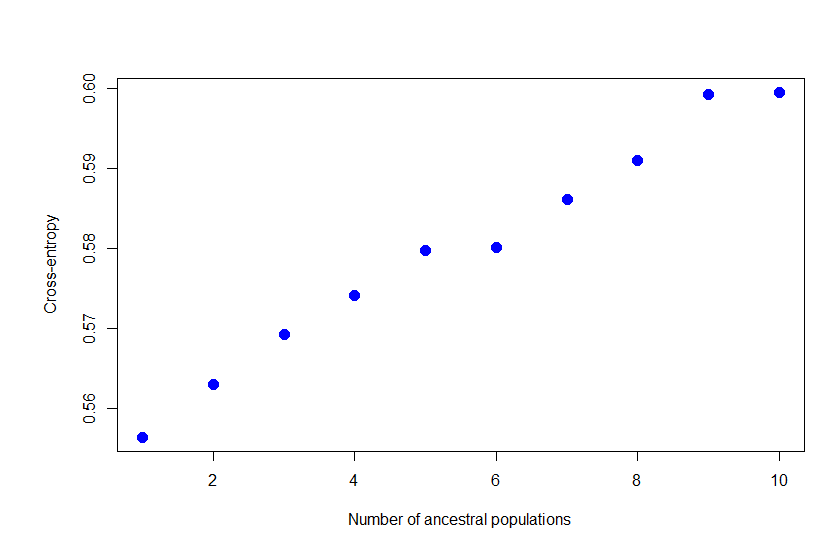


**Figure 4.** Minimal cross entropy for each number of ancestral populations (*K*) from 1 to 10, with 100 replications for each value of *K*. The value of *K* that best represents the population history is shown in red, and the value of K we used for the admixture proportions is shown in green. We chose to perform the analysis with a *K* value of 2 so that admixture proportions could be meaningfully visualised. Results from sNMF analysis of the DArTseq™ data filtered with a minor allele frequency of 0.02.


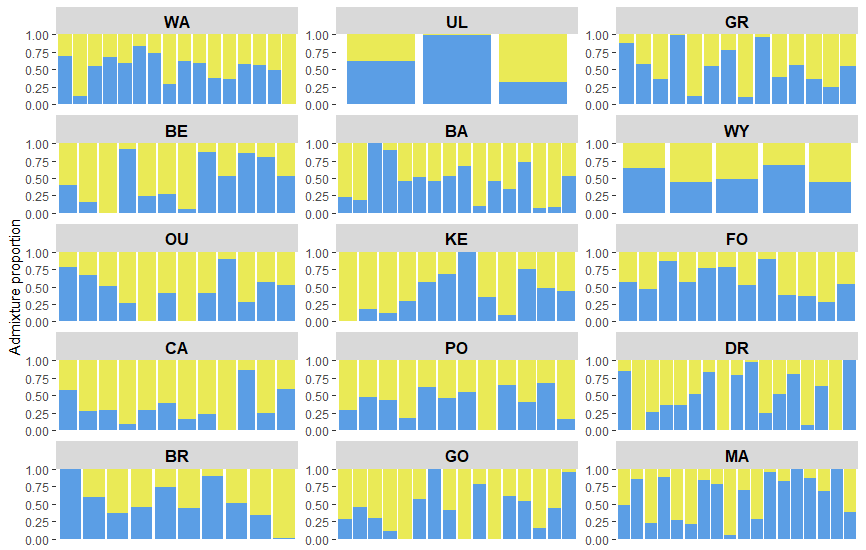

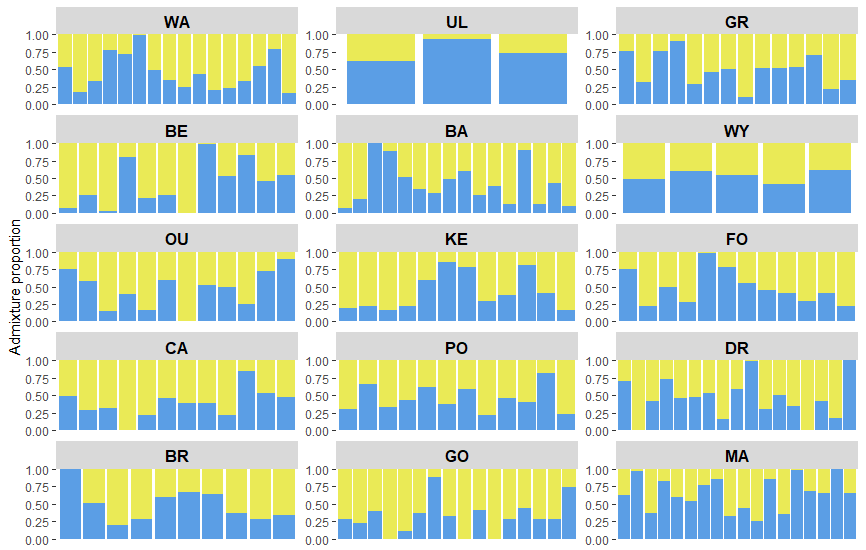


**(a)**

**(b)**


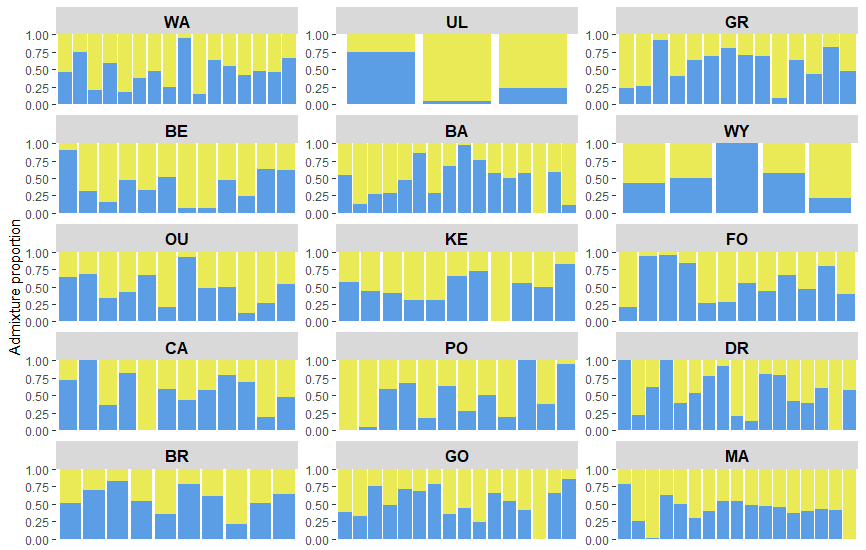


**(c)**

**Figure 5.** Comparisons of admixture proportions calculated after two different filtering methods based on DArTseq™ SNP data, as well as a different assembly method based on IPYRAD data: (a) DArTseq™ data filtered with a minor allele frequency of 0.02. (b) DArTseq™ data filtered with a minor allele count of 3, as has been recommended in previous studies (Linck and Battey et al. 2019; Hoffmann et al. 2021). (c) IPYRAD data filtered with a minor allele frequency of 0.05. Letters in bold represent assigned populations of *Chrysomya latifrons* (Diptera: Calliphoridae). Each bar represents a single individual.

**Table 1.** Results of divMigrate analysis. Numbers represent effective number of migrants (*Nm*). No significant asymmetries in gene flow were observed between populations.

|  | **BA** | **BE** | **BR** | **FO** | **GO** | **GR** | **MA** | **CA** | **DR** | **KE** | **OU** | **PO** | **UL** | **WA** | **WY** |
| --- | --- | --- | --- | --- | --- | --- | --- | --- | --- | --- | --- | --- | --- | --- | --- |
| **BA** | - | 0.7 | 0.6 | 0.7 | 0.8 | 0.8 | 1.0 | 0.7 | 1.0 | 0.7 | 0.7 | 0.7 | 0.2 | 0.9 | 0.3 |
| **BE** | 0.8 | - | 0.5 | 0.6 | 0.8 | 0.7 | 0.8 | 0.6 | 0.8 | 0.7 | 0.6 | 0.6 | 0.2 | 0.7 | 0.3 |
| **BR** | 0.7 | 0.6 | - | 0.6 | 0.6 | 0.6 | 0.7 | 0.5 | 0.7 | 0.6 | 0.6 | 0.6 | 0.2 | 0.7 | 0.3 |
| **FO** | 0.8 | 0.6 | 0.5 | - | 0.7 | 0.7 | 0.8 | 0.6 | 0.8 | 0.6 | 0.6 | 0.7 | 0.2 | 0.8 | 0.3 |
| **GO** | 0.9 | 0.7 | 0.6 | 0.7 | - | 0.8 | 0.9 | 0.7 | 0.9 | 0.7 | 0.7 | 0.7 | 0.2 | 0.8 | 0.3 |
| **GR** | 0.8 | 0.7 | 0.6 | 0.7 | 0.8 | - | 0.9 | 0.7 | 0.9 | 0.7 | 0.7 | 0.7 | 0.2 | 0.8 | 0.3 |
| **MA** | 1.0 | 0.7 | 0.6 | 0.8 | 0.9 | 0.8 | - | 0.7 | 1.0 | 0.7 | 0.7 | 0.8 | 0.2 | 0.9 | 0.3 |
| **CA** | 0.7 | 0.6 | 0.5 | 0.6 | 0.7 | 0.7 | 0.8 | - | 0.8 | 0.6 | 0.6 | 0.6 | 0.2 | 0.7 | 0.3 |
| **DR** | 0.9 | 0.8 | 0.6 | 0.8 | 0.9 | 0.8 | 1.0 | 0.7 | - | 0.8 | 0.7 | 0.8 | 0.2 | 0.9 | 0.3 |
| **KE** | 0.7 | 0.6 | 0.5 | 0.6 | 0.8 | 0.7 | 0.8 | 0.6 | 0.8 | - | 0.6 | 0.6 | 0.2 | 0.7 | 0.3 |
| **OU** | 0.8 | 0.6 | 0.5 | 0.6 | 0.8 | 0.7 | 0.8 | 0.6 | 0.8 | 0.7 | - | 0.7 | 0.2 | 0.7 | 0.3 |
| **PO** | 0.7 | 0.6 | 0.5 | 0.6 | 0.7 | 0.7 | 0.8 | 0.6 | 0.8 | 0.6 | 0.6 | - | 0.2 | 0.7 | 0.3 |
| **UL** | 0.2 | 0.2 | 0.2 | 0.2 | 0.2 | 0.2 | 0.2 | 0.2 | 0.2 | 0.2 | 0.2 | 0.2 | - | 0.2 | 0.1 |
| **WA** | 0.9 | 0.7 | 0.6 | 0.7 | 0.8 | 0.8 | 0.9 | 0.7 | 0.9 | 0.7 | 0.7 | 0.7 | 0.2 | - | 0.3 |
| **WY** | 0.4 | 0.3 | 0.3 | 0.4 | 0.4 | 0.4 | 0.4 | 0.3 | 0.4 | 0.4 | 0.4 | 0.3 | 0.1 | 0.4 | - |

**Table 2.** Raw specimen data and locality information. Only a subset of these specimens were included for the DArTseq™ sequencing.

| **Specimen ID** | **Population** | **Latitude** | **Longitude** | **Sex** |
| --- | --- | --- | --- | --- |
| L0001-MtKeira_Female | MtKeira | -34.4047 | 150.8713 | Female |
| L0002-MtKeira_Female | MtKeira | -34.4047 | 150.8713 | Female |
| L0003-MtKeira_Female | MtKeira | -34.4047 | 150.8713 | Female |
| L0004-MtKeira_Female | MtKeira | -34.4047 | 150.8713 | Female |
| L0005-MtKeira_Female | MtKeira | -34.4047 | 150.8713 | Female |
| L0006-MtKeira_Female | MtKeira | -34.4047 | 150.8713 | Female |
| L0012-MtKeira_Male | MtKeira | -34.4047 | 150.8713 | Male |
| L0013-MtKeira_Male | MtKeira | -34.4047 | 150.8713 | Male |
| L0014-MtKeira_Male | MtKeira | -34.4047 | 150.8713 | Male |
| L0015-MtKeira_Male | MtKeira | -34.4047 | 150.8713 | Male |
| L0016-MtKeira_Male | MtKeira | -34.4047 | 150.8713 | Male |
| L0017-MtKeira_Male | MtKeira | -34.4047 | 150.8713 | Male |
| L0031-Foxground_Female | Foxground | -34.6982 | 150.792 | Female |
| L0032-Foxground_Female | Foxground | -34.6982 | 150.792 | Female |
| L0033-Foxground_Female | Foxground | -34.6982 | 150.792 | Female |
| L0034-Foxground_Female | Foxground | -34.6982 | 150.792 | Female |
| L0035-Foxground_Female | Foxground | -34.6982 | 150.792 | Female |
| L0036-Foxground_Female | Foxground | -34.6982 | 150.792 | Female |
| L0041-Foxground_Male | Foxground | -34.6982 | 150.792 | Male |
| L0042-Foxground_Male | Foxground | -34.6982 | 150.792 | Male |
| L0043-Foxground_Male | Foxground | -34.6982 | 150.792 | Male |
| L0044-Foxground_Male | Foxground | -34.6982 | 150.792 | Male |
| L0045-Foxground_Male | Foxground | -34.6982 | 150.792 | Male |
| L0046-Foxground_Male | Foxground | -34.6982 | 150.792 | Male |
| L0074-MtCambewarra_Male | MtCambewarra | -34.8049 | 150.5722 | Male |
| L0075-MtCambewarra_Male | MtCambewarra | -34.8049 | 150.5722 | Male |
| L0076-MtCambewarra_Male | MtCambewarra | -34.8049 | 150.5722 | Male |
| L0077-MtCambewarra_Male | MtCambewarra | -34.8049 | 150.5722 | Male |
| L0078-MtCambewarra_Male | MtCambewarra | -34.8049 | 150.5722 | Male |
| L0079-MtCambewarra_Male | MtCambewarra | -34.8049 | 150.5722 | Male |
| L0097-MtCambewarra_Female | MtCambewarra | -34.8049 | 150.5722 | Female |
| L0098-MtCambewarra_Female | MtCambewarra | -34.8049 | 150.5722 | Female |
| L0099-MtCambewarra_Female | MtCambewarra | -34.8049 | 150.5722 | Female |
| L0100-MtCambewarra_Female | MtCambewarra | -34.8049 | 150.5722 | Female |
| L0101-MtCambewarra_Female | MtCambewarra | -34.8049 | 150.5722 | Female |
| L0102-MtCambewarra_Female | MtCambewarra | -34.8049 | 150.5722 | Female |
| L0115-PointerGap_Male | PointerGap | -35.2605 | 150.3582 | Male |
| L0116-PointerGap_Male | PointerGap | -35.2605 | 150.3582 | Male |
| L0117-PointerGap_Male | PointerGap | -35.2605 | 150.3582 | Male |
| L0118-PointerGap_Male | PointerGap | -35.2605 | 150.3582 | Male |
| L0119-PointerGap_Male | PointerGap | -35.2605 | 150.3582 | Male |
| L0120-PointerGap_Male | PointerGap | -35.2605 | 150.3582 | Male |
| L0142-PointerGap_Female | PointerGap | -35.2605 | 150.3582 | Female |
| L0143-PointerGap_Female | PointerGap | -35.2605 | 150.3582 | Female |
| L0144-PointerGap_Female | PointerGap | -35.2605 | 150.3582 | Female |
| L0145-PointerGap_Female | PointerGap | -35.2605 | 150.3582 | Female |
| L0146-PointerGap_Female | PointerGap | -35.2605 | 150.3582 | Female |
| L0147-PointerGap_Female | PointerGap | -35.2605 | 150.3582 | Female |
| L0151-BarringtonTops_Male | BarringtonTops | -32.1508 | 151.5248 | Male |
| L0152-BarringtonTops_Male | BarringtonTops | -32.1508 | 151.5248 | Male |
| L0153-BarringtonTops_Male | BarringtonTops | -32.1508 | 151.5248 | Male |
| L0154-BarringtonTops_Male | BarringtonTops | -32.1508 | 151.5248 | Male |
| L0155-BarringtonTops_Male | BarringtonTops | -32.1508 | 151.5248 | Male |
| L0156-BarringtonTops_Male | BarringtonTops | -32.1508 | 151.5248 | Male |
| L0157-BarringtonTops_Male | BarringtonTops | -32.1508 | 151.5248 | Male |
| L0158-BarringtonTops_Male | BarringtonTops | -32.1508 | 151.5248 | Male |
| L0171-BarringtonTops_Female | BarringtonTops | -32.1508 | 151.5248 | Female |
| L0172-BarringtonTops_Female | BarringtonTops | -32.1508 | 151.5248 | Female |
| L0173-BarringtonTops_Female | BarringtonTops | -32.1508 | 151.5248 | Female |
| L0174-BarringtonTops_Female | BarringtonTops | -32.1508 | 151.5248 | Female |
| L0175-BarringtonTops_Female | BarringtonTops | -32.1508 | 151.5248 | Female |
| L0176-BarringtonTops_Female | BarringtonTops | -32.1508 | 151.5248 | Female |
| L0177-BarringtonTops_Female | BarringtonTops | -32.1508 | 151.5248 | Female |
| L0178-BarringtonTops_Female | BarringtonTops | -32.1508 | 151.5248 | Female |
| L0207-UlidarraNP_Male | UlidarraNP | -30.2488 | 153.0855 | Male |
| L0208-UlidarraNP_Male | UlidarraNP | -30.2488 | 153.0855 | Male |
| L0209-UlidarraNP_Male | UlidarraNP | -30.2488 | 153.0855 | Male |
| L0210-Bellangry_Male | Bellangry | -31.2892 | 152.537 | Male |
| L0211-Bellangry_Male | Bellangry | -31.2892 | 152.537 | Male |
| L0212-Bellangry_Male | Bellangry | -31.2892 | 152.537 | Male |
| L0213-Bellangry_Male | Bellangry | -31.2892 | 152.537 | Male |
| L0214-Bellangry_Male | Bellangry | -31.2892 | 152.537 | Male |
| L0215-Bellangry_Male | Bellangry | -31.2892 | 152.537 | Male |
| L0239-Bellangry_Male | Bellangry | -31.2892 | 152.537 | Male |
| L0240-Bellangry_Female | Bellangry | -31.2892 | 152.537 | Female |
| L0241-Bellangry_Female | Bellangry | -31.2892 | 152.537 | Female |
| L0242-Bellangry_Female | Bellangry | -31.2892 | 152.537 | Female |
| L0243-Bellangry_Female | Bellangry | -31.2892 | 152.537 | Female |
| L0244-Bellangry_Female | Bellangry | -31.2892 | 152.537 | Female |
| L0249-Ourimbah_Male | Ourimbah | -33.3649 | 151.3939 | Male |
| L0250-Ourimbah_Male | Ourimbah | -33.3649 | 151.3939 | Male |
| L0251-Ourimbah_Male | Ourimbah | -33.3649 | 151.3939 | Male |
| L0252-Ourimbah_Male | Ourimbah | -33.3649 | 151.3939 | Male |
| L0253-Ourimbah_Male | Ourimbah | -33.3649 | 151.3939 | Male |
| L0254-Ourimbah_Male | Ourimbah | -33.3649 | 151.3939 | Male |
| L0267-Ourimbah_Female | Ourimbah | -33.3649 | 151.3939 | Female |
| L0268-Ourimbah_Female | Ourimbah | -33.3649 | 151.3939 | Female |
| L0269-Ourimbah_Female | Ourimbah | -33.3649 | 151.3939 | Female |
| L0270-Ourimbah_Female | Ourimbah | -33.3649 | 151.3939 | Female |
| L0271-Ourimbah_Female | Ourimbah | -33.3649 | 151.3939 | Female |
| L0272-Ourimbah_Female | Ourimbah | -33.3649 | 151.3939 | Female |
| L0276-WyrrabalongNP_Male | WyrrabalongNP | -33.2938 | 151.5356 | Male |
| L0277-WyrrabalongNP_Male | WyrrabalongNP | -33.2938 | 151.5356 | Male |
| L0278-WyrrabalongNP_Male | WyrrabalongNP | -33.2938 | 151.5356 | Male |
| L0279-WyrrabalongNP_Male | WyrrabalongNP | -33.2938 | 151.5356 | Male |
| L0280-WyrrabalongNP_Male | WyrrabalongNP | -33.2938 | 151.5356 | Male |
| L0281-GrahamsTrail_Male | GrahamsTrail | -30.4245 | 152.8304 | Male |
| L0282-GrahamsTrail_Male | GrahamsTrail | -30.4245 | 152.8304 | Male |
| L0283-GrahamsTrail_Male | GrahamsTrail | -30.4245 | 152.8304 | Male |
| L0284-GrahamsTrail_Male | GrahamsTrail | -30.4245 | 152.8304 | Male |
| L0285-GrahamsTrail_Male | GrahamsTrail | -30.4245 | 152.8304 | Male |
| L0286-GrahamsTrail_Male | GrahamsTrail | -30.4245 | 152.8304 | Male |
| L0287-GrahamsTrail_Male | GrahamsTrail | -30.4245 | 152.8304 | Male |
| L0291-GrahamsTrail_Female | GrahamsTrail | -30.4245 | 152.8304 | Female |
| L0292-GrahamsTrail_Female | GrahamsTrail | -30.4245 | 152.8304 | Female |
| L0293-GrahamsTrail_Female | GrahamsTrail | -30.4245 | 152.8304 | Female |
| L0294-GrahamsTrail_Female | GrahamsTrail | -30.4245 | 152.8304 | Female |
| L0295-GrahamsTrail_Female | GrahamsTrail | -30.4245 | 152.8304 | Female |
| L0296-GrahamsTrail_Female | GrahamsTrail | -30.4245 | 152.8304 | Female |
| L0297-GrahamsTrail_Female | GrahamsTrail | -30.4245 | 152.8304 | Female |
| L0298-Washpool_Male | Washpool | -29.47 | 152.316 | Male |
| L0299-Washpool_Male | Washpool | -29.47 | 152.316 | Male |
| L0300-Washpool_Male | Washpool | -29.47 | 152.316 | Male |
| L0301-Washpool_Male | Washpool | -29.47 | 152.316 | Male |
| L0302-Washpool_Male | Washpool | -29.47 | 152.316 | Male |
| L0304-Washpool_Male | Washpool | -29.47 | 152.316 | Male |
| L0305-Washpool_Male | Washpool | -29.47 | 152.316 | Male |
| L0306-Washpool_Male | Washpool | -29.47 | 152.316 | Male |
| L0327-Washpool_Female | Washpool | -29.47 | 152.316 | Female |
| L0328-Washpool_Female | Washpool | -29.47 | 152.316 | Female |
| L0329-Washpool_Female | Washpool | -29.47 | 152.316 | Female |
| L0330-Washpool_Female | Washpool | -29.47 | 152.316 | Female |
| L0331-Washpool_Female | Washpool | -29.47 | 152.316 | Female |
| L0332-Washpool_Female | Washpool | -29.47 | 152.316 | Female |
| L0333-Washpool_Female | Washpool | -29.47 | 152.316 | Female |
| L0334-Washpool_Female | Washpool | -29.47 | 152.316 | Female |
| L0335-MtDromedary_Male | MtDromedary | -36.2946 | 150.0337 | Male |
| L0336-MtDromedary_Male | MtDromedary | -36.2946 | 150.0337 | Male |
| L0337-MtDromedary_Male | MtDromedary | -36.2946 | 150.0337 | Male |
| L0338-MtDromedary_Male | MtDromedary | -36.2946 | 150.0337 | Male |
| L0339-MtDromedary_Male | MtDromedary | -36.2946 | 150.0337 | Male |
| L0340-MtDromedary_Male | MtDromedary | -36.2946 | 150.0337 | Male |
| L0341-MtDromedary_Male | MtDromedary | -36.2946 | 150.0337 | Male |
| L0342-MtDromedary_Male | MtDromedary | -36.2946 | 150.0337 | Male |
| L0350-MtDromedary_Female | MtDromedary | -36.2946 | 150.0337 | Female |
| L0351-MtDromedary_Female | MtDromedary | -36.2946 | 150.0337 | Female |
| L0352-MtDromedary_Female | MtDromedary | -36.2946 | 150.0337 | Female |
| L0353-MtDromedary_Female | MtDromedary | -36.2946 | 150.0337 | Female |
| L0354-MtDromedary_Female | MtDromedary | -36.2946 | 150.0337 | Female |
| L0355-MtDromedary_Female | MtDromedary | -36.2946 | 150.0337 | Female |
| L0356-MtDromedary_Female | MtDromedary | -36.2946 | 150.0337 | Female |
| L0357-MtDromedary_Female | MtDromedary | -36.2946 | 150.0337 | Female |
| L0358-MtDromedary_Female | MtDromedary | -36.2946 | 150.0337 | Female |
| L0361-BrownMountain_Male | BrownMountain | -36.5972 | 149.444 | Male |
| L0362-BrownMountain_Male | BrownMountain | -36.5972 | 149.444 | Male |
| L0363-BrownMountain_Male | BrownMountain | -36.5972 | 149.444 | Male |
| L0364-BrownMountain_Male | BrownMountain | -36.5972 | 149.444 | Male |
| L0365-BrownMountain_Male | BrownMountain | -36.5972 | 149.444 | Male |
| L0366-BrownMountain_Male | BrownMountain | -36.5972 | 149.444 | Male |
| L0367-BrownMountain_Male | BrownMountain | -36.5972 | 149.444 | Male |
| L0368-BrownMountain_Male | BrownMountain | -36.5972 | 149.444 | Male |
| L0369-BrownMountain_Female | BrownMountain | -36.5972 | 149.444 | Male |
| L0370-BrownMountain_Female | BrownMountain | -36.5972 | 149.444 | Male |
| L0371-MaxwellsRainforest_Male | MaxwellsRainforest | -37.4145 | 149.8138 | Male |
| L0372-MaxwellsRainforest_Male | MaxwellsRainforest | -37.4145 | 149.8138 | Male |
| L0373-MaxwellsRainforest_Male | MaxwellsRainforest | -37.4145 | 149.8138 | Male |
| L0374-MaxwellsRainforest_Male | MaxwellsRainforest | -37.4145 | 149.8138 | Male |
| L0375-MaxwellsRainforest_Male | MaxwellsRainforest | -37.4145 | 149.8138 | Male |
| L0376-MaxwellsRainforest_Male | MaxwellsRainforest | -37.4145 | 149.8138 | Male |
| L0377-MaxwellsRainforest_Male | MaxwellsRainforest | -37.4145 | 149.8138 | Male |
| L0378-MaxwellsRainforest_Male | MaxwellsRainforest | -37.4145 | 149.8138 | Male |
| L0379-MaxwellsRainforest_Male | MaxwellsRainforest | -37.4145 | 149.8138 | Male |
| L0385-MaxwellsRainforest_Female | MaxwellsRainforest | -37.4145 | 149.8138 | Female |
| L0386-MaxwellsRainforest_Female | MaxwellsRainforest | -37.4145 | 149.8138 | Female |
| L0387-MaxwellsRainforest_Female | MaxwellsRainforest | -37.4145 | 149.8138 | Female |
| L0388-MaxwellsRainforest_Female | MaxwellsRainforest | -37.4145 | 149.8138 | Female |
| L0389-MaxwellsRainforest_Female | MaxwellsRainforest | -37.4145 | 149.8138 | Female |
| L0390-MaxwellsRainforest_Female | MaxwellsRainforest | -37.4145 | 149.8138 | Female |
| L0391-MaxwellsRainforest_Female | MaxwellsRainforest | -37.4145 | 149.8138 | Female |
| L0392-MaxwellsRainforest_Female | MaxwellsRainforest | -37.4145 | 149.8138 | Female |
| L0393-MaxwellsRainforest_Female | MaxwellsRainforest | -37.4145 | 149.8138 | Female |
| L0394-GoodeniaRainforest_Male | GoodeniaRainforest | -36.899 | 149.7154 | Male |
| L0395-GoodeniaRainforest_Male | GoodeniaRainforest | -36.899 | 149.7154 | Male |
| L0396-GoodeniaRainforest_Male | GoodeniaRainforest | -36.899 | 149.7154 | Male |
| L0397-GoodeniaRainforest_Male | GoodeniaRainforest | -36.899 | 149.7154 | Male |
| L0398-GoodeniaRainforest_Male | GoodeniaRainforest | -36.899 | 149.7154 | Male |
| L0399-GoodeniaRainforest_Male | GoodeniaRainforest | -36.899 | 149.7154 | Male |
| L0400-GoodeniaRainforest_Male | GoodeniaRainforest | -36.899 | 149.7154 | Male |
| L0401-GoodeniaRainforest_Male | GoodeniaRainforest | -36.899 | 149.7154 | Male |
| L0402-GoodeniaRainforest_Male | GoodeniaRainforest | -36.899 | 149.7154 | Male |
| L0411-GoodeniaRainforest_Female | GoodeniaRainforest | -36.899 | 149.7154 | Female |
| L0412-GoodeniaRainforest_Female | GoodeniaRainforest | -36.899 | 149.7154 | Female |
| L0413-GoodeniaRainforest_Female | GoodeniaRainforest | -36.899 | 149.7154 | Female |
| L0414-GoodeniaRainforest_Female | GoodeniaRainforest | -36.899 | 149.7154 | Female |
| L0415-GoodeniaRainforest_Female | GoodeniaRainforest | -36.899 | 149.7154 | Female |
| L0416-GoodeniaRainforest_Female | GoodeniaRainforest | -36.899 | 149.7154 | Female |
| L0417-GoodeniaRainforest_Female | GoodeniaRainforest | -36.899 | 149.7154 | Female |
